# Supplementary material for: Optimized residue analysis method for broflanilide and its metabolites in agricultural produce using the QuEChERS method and LC-MS/MS
Source: PLoS One. 2020 Oct 6;15(10):e0235526. doi: 10.1371/journal.pone.0235526 (PMC7537868; doi:10.1371/journal.pone.0235526)
Supplement: Attachment — Submitted filename: Response to reviewers.docx [file pone.0235526.s001.docx]

**Response to Reviewers**

**Reviewer 1.**

**Comment 1.** Why didn’t you include the internal standard?

**Answer:** Internal standards are used to reduce the matrix effect. However, the matrix effect observed in our study was insignificant, i.e., did not have to be reduced via internal standard addition. In the future, with the above comment in mind, we will investigate the effects of internal standard addition.

**Comment 2.** There is little matrix effect, so you don’t have to report it.

**Answer:** In most cases, the matrix effect was low, but in some cases it was moderate. Therefore, we provided results for various cases to suggest that an appropriate method can be selected for the analysis of broflanilide and its metabolites in agricultural products.

**Reviewer 2.**

**Comment 1.** It is recommended to present the analysis results by applying the established analysis method to the real samples.

**Answer:** We totally agree with this comment. Agricultural products in circulation in Korea are monitored by certain institutions for pesticide residues. In particular, our agency aims to develop analytical methods (such as that described in the present work) that can be used by these institutions. So, we not carried out to monitor pesticide residue in circulating agricultural products.

**Editor comments**

**Comment 1.** Please ensure that your manuscript meets PLOS ONE's style requirements, including those for file naming.

**Answer:** The necessary changes have been made: the excessive use of acronyms has been avoided, references have been formatted, etc.

**Comment 2.** In your Methods, please state the exact source of the samples used in your study.

**Answer:** The untreated samples were purchased from an environmentally friendly agricultural produce market, Chorocmaeul (www.choroc.com) in Wanju, Korea. (Page5, Line100)

**Comment 3.** PLOS requires an ORCID iD for the corresponding author in Editorial Manager on papers submitted after December 6th, 2016. Please ensure that you have an ORCID iD and that it is validated in Editorial Manager.

**Answer:** An ORCID iD for the corresponding author has been successfully created and validated.
